# Determinants of Folate and Vitamin B_12_ Deficiencies in Women of Reproductive Age: Insights from the 2018 National Nutrition Survey of Pakistan

**DOI:** 10.3390/nu18071128

**Published:** 2026-03-31

**Authors:** Junaid Iqbal, Kehkashan Begum, Rabia Zuberi, Muhammad Sajid, Sidrah Nausheen, Imran A. Chauhadry, Sajid Bashir Soofi, Zulfiqar A. Bhutta

**Affiliations:** 1 Department of Pediatrics and Child Health, Aga Khan University, Stadium Road, Karachi 74800, Pakistan; junaid.iqbal@aku.edu (J.I.);; 2 Center of Excellence for Women and Children, Aga Khan University, Stadium Road, Karachi 74800, Pakistan; 3 Institute for Global Health & Development, Aga Khan University, Stadium Road, Karachi 74800, Pakistan

**Keywords:** women of reproductive age, B_12_ deficiency, folate deficiency, Pakistan

## Abstract

**Background:** Anemia is a major public health issue, particularly among women of reproductive age (WRA) in low- and middle-income countries (LMICs). Pakistan’s National Nutrition Survey (NNS) 2011 showed a high prevalence of vitamin B_12_ (B_12_) and folate deficiency among WRA, necessitating further investigation in subsequent surveys. **Methods:** Blood samples from 31,828 WRA (15–49 years old) were collected using a stratified multi-stage sampling technique in NNS-2018. We conducted a secondary analysis using population-weighted logistic regression to assess the association of potential factors with B_12_ and folate deficiency. B_12_ (*n* = 4442) and folate (*n* = 12,662) samples were measured using an electrochemiluminescence immunoassay and a Centers for Disease Control and Prevention, USA (CDC)-approved microbiologic assay, respectively. **Results:** Folate deficiency was present in 44.7% WRA, and 20.2% had B_12_ deficiency. Provincial distribution was associated with folate deficiency, i.e., Sindh (OR = 1.140, 95% CI 1.018, 1.285), Baluchistan (OR = 1.237, 95% CI 1.052, 1.453), and Islamabad (OR = 1.524, 95% CI 1.109, 2.092), while B_12_ deficiency was prevalent in Islamabad (OR = 1.673, 95% CI 1.122, 2.497), Gilgit Baltistan (OR = 2.472, 95% CI 1.197, 5.106), and newly merged districts of KPK (OR = 1.584, 95% CI 0.977, 2.570). Rural residence (OR = 1.407, 95% CI 1.125, 1.760), obesity (OR = 1.649, 95% CI 1.282, 2.122), and overweight (OR = 1.560, 95% CI 1.262, 1.928) were associated with B_12_ deficiency. **Conclusions:** Our results show regional and demographic differences in the prevalence of folate and B_12_ deficiencies among WRA. This underscores the need for targeted nutritional interventions and further longitudinal studies to identify potentially associated factors.

## 1. Background

Micronutrient deficiencies are a critical public health issue globally, particularly among WRA who have higher nutrient needs than other groups and face greater socioeconomic and biological risks [1]. Adequate micronutrient levels are essential for this population, as deficiency can harm women’s health and affect fetal development during pregnancy [2]. Of all the micronutrients, B_12_ and folate are considered the most critical for maintaining an adequate status among WRA, as their deficiency can negatively affect maternal health and reproductive functions [3]. Despite their importance and being biologically vital, B_12_ and folate deficiencies still occur frequently in WRA globally, indicating that there are still considerable shortcomings in their diets, as well as in the intake of these micronutrients [4,5]. Additionally, there is a link between low B_12_ levels during pregnancy and a higher chance of adverse pregnancy outcomes [6]. Folate deficiency has also been associated with atherosclerosis, stunting, and an increased risk of low birth weight and neural tube closure defects, including encephalocele, anencephaly, and spina bifida, during pregnancy [7,8]. On the other hand, B_12_ and folate deficits are linked to megaloblastic anemia, and both vitamins are involved in a variety of common metabolic processes [9]. Folate deficiency leads to blood changes that are identical to those caused by a lack of B_12_, and their deficiencies often occur together, ultimately increasing the likelihood of anemia [10]. Anemia is a serious global public health issue that affects both developed and developing countries and is more common in young children, adolescent girls, women of reproductive age, pregnant women, and postpartum women [11]. Left untreated, anemia can result in fatigue, limit daily activities, and potentially cause heart problems [12] or pregnancy complications [13]. Anemia in women during their reproductive age has been associated with impaired ovulation, disturbed endometrial receptivity, and menstrual irregularities [14,15]. In Pakistan, among WRA, the prevalence of anemia was 44% in pregnant women compared to 41.1% in non-pregnant women, according to the World Health Organization (WHO) in 2019 [16]. Although anemia is mostly evaluated by measuring hemoglobin levels, serving as a sign of iron deficiency, in the past, national programs for the control of anemia in Pakistan have mostly given priority to iron deficiency and hemoglobin-based screening. But, as iron deficiency is not the sole reason for anemia, deficiencies in B_12_ and folate can also lead to this condition. Therefore, this emphasizes the importance of measuring these vitamins for identifying vitamin-associated megaloblastic anemia [17].

B_12_ and folate deficiencies can be effectively addressed through combined dietary supplementation and public health strategies, such as screening high-risk populations for micronutrient deficiencies, using biomarker monitoring alongside nutrition education, and promoting dietary diversity. Primary dietary sources of folate include plant foods such as leafy greens and fruits, as well as animal foods such as yeast, meat, and liver. Animals’ gut microbes produce B_12_, which is then absorbed and integrated into animal tissues. Therefore, the only source of B_12_ for humans is products from herbivorous animals, such as meat, eggs, and milk. Thus, the risk of folate and B_12_ deficiency is increased when there is an inadequate intake of these foods [18].

We evaluated the association between B_12_ and folate deficiencies among Pakistani women and their contributing factors, using data from Pakistan’s 2011 National Nutrition Survey (NNS). This survey indicated that 52.4% of WRA had B_12_ deficiency and 50.8% had folate deficiency. The survey found a high prevalence of anemia (50.4%) in the region and reported a significant positive correlation with B_12_ deficiency in two provinces. In Khyber Pakhtunkhwa, the risk of being B_12_ deficient was 1.25 (95% CI 1.11 to 1.43), *p* < 0.00, while Azad Jammu and Kashmir showed an even greater risk, with an RR of 1.50 (95% CI 1.08 to 2.08), *p* = 0.01 [19]. Following NNS-2011, studies have investigated the supply, demand, perceptions, knowledge, and practices regarding iron and folic acid (IFA) supplements in rural and urban settings in Pakistan. Qualitative research that was conducted to assess the supply and demand of maternal iron–folic acid has reported that IFA supplements are mostly available to pregnant women via the LHW network, district clinics, and private pharmacies [20].

Our analysis is based on data from the very recent Pakistan National Nutrition Survey (NNS) 2018, which provides updated estimates of folic acid and B_12_ deficiencies among reproductive-aged women in Pakistan, as there have been considerable changes in the population, nutrition, and programs since the NNS-2011. The previous analysis, based on NNS-2011 data published in 2017, established the baseline for WRA’s micronutrient deficiencies. However, our present study does not merely confirm the previous findings, but goes a step further by employing a new analytical framework that considers a broader range of sociodemographic, dietary, and environmental factors, such as sanitation, drinking water sources, and wealth index. Therefore, this analysis seeks to look beyond the prevalence estimates and explore the possible associated factors of micronutrient deficiencies in the country to contribute to the generation of evidence that could be useful in improving nutritional and public health strategies. This also gives the opportunity to evaluate the current risk profiles and changes in deficiency rates, with respect to the recent nutrition strategies, fortification programs, and maternal healthcare programs implemented over the last decade.

## 2. Materials and Methods

### 2.1. Study Design

The National Nutrition Survey (NNS) April 2018–January 2019 was a cross-sectional, household (HH)-based survey that was conducted to measure nutrition indicators in Pakistan. Both qualitative and quantitative research techniques were used to gather substantial data. The sampling frame was designed to obtain the district-level data of all provinces of Pakistan, including Punjab, Sindh, Baluchistan, Khyber Pakhtunkhwa (KPK), Azad Jammu and Kashmir (AJK), Gilgit-Baltistan (GB), Khyber Pakhtunkhwa-Newly Merged Districts (KP-NMD), and Islamabad Capital Territory (ICT) from rural/urban localities for both genders.

### 2.2. Sample Size Calculation

We used WRA data from the National Nutrition Survey (NNS) of Pakistan 2011 to determine the sample size needed to estimate the prevalence of folate and B_12_ deficiencies in Pakistan. To account for multi-stage cluster sampling, the estimates were calculated using a design effect of two and an alpha level of 5%. It would take at least 541 participants to estimate the folate and 540 people to estimate the B_12_ deficiency among WRA at a 90% confidence level. The following formula has been used to determine the sample size.

Sample size (*n*) = [DEFF × Np(1 − p)]/[d^2^/Z^2^_1−α/2_ × (N − 1) + p × (1 − p)]

### 2.3. Sampling Technique

The Population and Housing Census 2017 served as the basis for the sample frame provided by the Pakistan Bureau of Statistics (PBS), which also produced digital maps of prominent localities and compact enumeration blocks, or Primary Sampling Units (PSUs), with 200–250 households. At the household level, 115,600 secondary sampling units (SSUs) were chosen from 5780 PSUs. The line-listing exercise from Aga Khan University (AKU) was used to nominate households from each sampled PSU by the field personnel. AKU’s Data Management Unit (DMU) collected paper-based line-listing data and entered it into electronic tablets to select the study population.

For this analysis, we adopted a pragmatic, availability-based sampling approach. Folate and B_12_ analyses were done on all eligible archived plasma samples with sufficient residual volume.

### 2.4. Blood Collection

Venous blood samples were collected from participants by qualified phlebotomists while following the WHO safe phlebotomy guidelines [21]. During the NNS-2018 survey, 31,828 women of reproductive age (WRA) aged 15–49 years provided blood samples.

**Figure 1:**
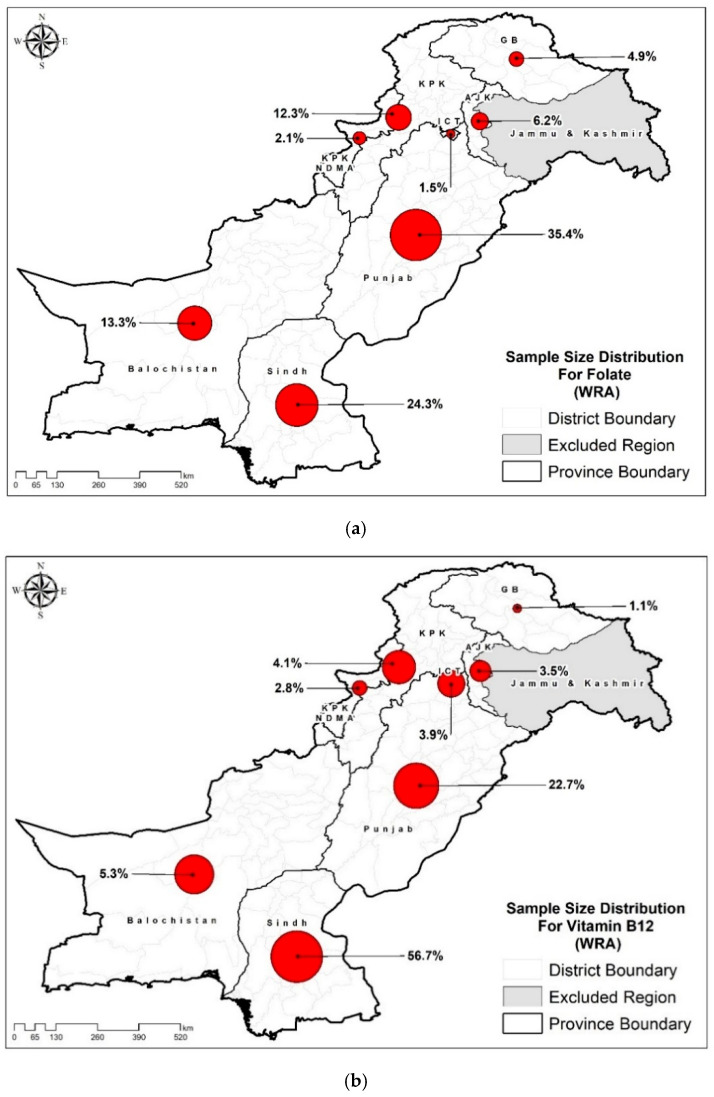
(**a**) Regional distribution of sampled population for plasma folate analysis. (**b**): Regional distribution of sampled population for plasma B_12_ analysis.

B_12_ and folate assays were performed on a subset, chosen from NNS-2018’s archived blood plasma samples. A total of 12,662 and 4442 samples were assayed to measure the folate and B_12_ concentrations, respectively (Figure 1).

### 2.5. Biochemical Assessment

Biochemical assessments were conducted in the Nutrition Research Laboratory (NRL) at AKU, Karachi, Pakistan. B_12_ was quantitatively measured in plasma samples using an electrochemiluminescence immunoassay (ECLIA), a competitive binding assay that employs intrinsic B_12_-specific factors for selective detection. The Roche Cobas e411 Vitamin B_12_ II assay kit (Reference # 07212771 190) was used for the analysis, with a detection range of 50 to 2000 pg/mL (Roche Diagnostics, Basel, Switzerland). Calibration was performed using a Roche Vitamin B_12_ II Cal Set (Reference # 07212780 190), following a two-point calibration process once per reagent lot, within 24 h of reagent registration, every 28 days for the same lot, or at least once every 7 days when using the same reagent kit. To ensure accuracy, PreciControl Varia (Reference # 05618860 190) was used for quality control, with high- and low-concentration controls run once per batch-run, a new reagent kit, and after each calibration. The assay’s reliability was further confirmed through external validation in the CDC Vital External Quality Assurance (EQA) program, a standardization program that provides independent assessment of analytical performance for measuring different nutritional markers in plasma/serum samples [22]. We received three serum/plasma samples of an unknown concentration twice per year for external quality assurance. Results were compared for relative differences from the CDC’s target value and imprecision, using round-by-round descriptive statistics. Statistical parameters were used as a basis for the performance criteria and were considered to be acceptable (optimal, desirable, or minimal performance) or unacceptable (less than minimal performance). During the analysis of this survey, we participated in the CDC Vital EQA round 33. Assay performance showed measurements within ±17.2% of the target values with an analytical imprecision of <3.7%, with an overall acceptance of 100% for the assay.

Folate levels were analyzed using a microbiologic assay [23,24]. This gold-standard method is recommended by the CDC, USA, due to its accuracy and ability to detect all biologically active forms of folate, making it ideal for population surveys. The assay used 5-methyltetrahydrofolate as a calibrator for precise quantification. The kit provided a naturally auxotrophic strain of *Lactobacillus rhamnosus* resuspended in folate-free medium, ensuring that its proliferation depended solely on folate availability in the serum/plasma samples. To perform the assay, diluted plasma samples were added to 96-well plates containing the inoculated growth medium. The plates were incubated for 42 h at 37 °C, allowing the bacterial growth to reflect the folate concentration in the plasma samples. The samples were tested in quadruplicate to enhance precision, and the folate levels were determined by measuring the bacterial growth at an optical density of 590 nm, using a microplate reader. NRL staff received training from CDC Atlanta, with support from CDC’s Folate Task Team and Nutrition International, to ensure proper assay optimization at NRL. The method’s reliability was then validated through participating in CDC’s 2020 Method Performance Verification for serum folate and achieved an imprecision of <2.5% and a deviation of <0.27% from the target value, where the acceptable limits were <16.1% and <20.0%, respectively.

### 2.6. Study Variables

To evaluate B_12_ and folate deficiencies in WRA in Pakistan, covariates were selected based on their conceptual importance, prior research, and established factors for evaluating micronutrient status in LMICs. Sociodemographic variables included residence (urban/rural), province, household size, and household wealth index, as in Pakistan’s Demographic and Health Surveys (DHS) [25], as they may influence health outcomes. Water and sanitation conditions were evaluated based on drinking water sources and sanitation facilities, classified as improved or unimproved, according to the WHO/United Nations International Children’s Emergency Fund (UNICEF), Joint Monitoring Program (JMP) classifications. The use of these indicators reveals the extent of exposure to unsanitary conditions and poor hygiene, which can impact the susceptibility to infections and the health of women in their reproductive age [26]. Women’s education [27], occupation, age, parity [28], body mass index (BMI) [29], marital status, dietary diversity [30], and pregnancy status [31] were selected based on evidence for the association of socioeconomic status (SES) and reproductive characteristics with their overall health. Anemia status was classified according to pregnancy status, and pregnant WRAs were evaluated as anemic for a hemoglobin level of <11 g/dL, while non-pregnant WRAs with a hemoglobin level of <12 g/dL were considered anemic [32].

### 2.7. Statistical Analysis

Descriptive analysis was conducted to summarize the study population, presenting the percentage of individuals in each variable category, along with 95% confidence intervals (CIs). The folate and B_12_ concentrations were classified as the deficiency status (deficient/non-deficient) as per cut-offs established for the CDC’s microbiology assay, i.e., <7 nmoL/L (3 ng/mL) for serum/plasma folate levels [23] and <191 pg/mL for B_12_ levels that align with cut-offs commonly used in large-scale population surveys utilizing ECLIA [33]. The prevalence of deficiency was reported across study variables.

To identify factors associated with folate and B_12_ deficiency, logistic regression analysis was performed, while accounting for the complex survey design, including weighting and clustering at the enumeration block level. The sampling weights were applied, using the svy command in Stata to obtain population-representative estimates, and standard errors were calculated using the linearized variance estimation method by Taylor series linearization. The sampling weights were calculated as the inverse probability of selection using a two-stage design and adjusted for non-response. Descriptive estimates and 95% confidence intervals were obtained using survey-adjusted methods.

We used a forward stepwise modeling approach to identify potential predictors while reducing the number of variables in the final model. Variables with a *p*-value < 0.25 in the bivariate analysis were considered for inclusion in the multivariable model. Collinear variables (correlation coefficient > 0.8) were excluded, using correlation analysis, the Eta coefficient, and Cramér’s V, and a retention criterion of *p* < 0.10 was applied during adjusted analysis. Some important variables, including residence, SES, food insecurity status, and wealth index, were retained in the model regardless of their *p*-values. This approach has been used in epidemiological studies to ensure that potentially important variables are not excluded prematurely. Unadjusted and adjusted odds ratios (ORs and aORs), along with 95% Cis, have been reported for all the study variables. As a further test for models’ adequacy, the variances of inflation factors (VIF) were imposed on all variables. All statistical analyses were performed using Stata version 17.

## 3. Results

### 3.1. Characteristics of the Study Population

Data on sociodemographic, anthropometric, environmental food insecurity, and health indicators for the study population (WRA) are presented in Table 1. A total of 60% of WRA were from rural areas of Pakistan. Provincial distribution was skewed, with more WRAs from Sindh and Punjab: around 50% of folate samples were from Punjab, while 60% of B_12_ samples were from Sindh. Most of the samples were from WRA aged between 20 and 34 years. An almost equal number of samples were collected from each household-size category for both assays, while a slightly higher proportion of B_12_ samples came from the poorest households compared with other wealth index categories. The majority of the WRAs had improved drinking water sources, improved sanitation facilities, and access to secure food, but with insufficient dietary diversity. Similarly, a higher proportion of WRA had a normal BMI, ≥3 parity status, and no formal education. Most of the WRA at the time of sampling were non-pregnant (around 94%).

### 3.2. Prevalence of B_12_ and Folate Deficiency Among Women of Reproductive Age

Table 2 shows the distribution of the WRA population, based on plasma folate and B_12_ deficiency. Approximately 45% of the WRA were folate-deficient, regardless of whether they lived in urban or rural areas. Similar levels of folate deficiency were observed for WRA living in households with either <7 or ≥7 family members. All categories of wealth index, food insecurity status, dietary diversity, drinking water source and sanitation facility also showed folate deficiency in around 45% of the WRA. Approximately a similar proportion of women showed folate deficiency across different categories of education, occupation status, marital status, BMI, parity, and pregnancy status. Folate deficiency levels varied across provinces, with about 39.1% of women in Azad Jammu and Kashmir affected, while the highest prevalence was seen in Islamabad Capital Territory at 53.6%. Younger WRA (15 to 19 years old) also had a slightly higher prevalence, with around 51.3% affected by folate deficiency.

B_12_ deficiency was observed in a smaller proportion of WRA compared to folate deficiency, with slightly more rural women affected (21.8%) than urban women (17.8%). The prevalence of B_12_ deficiency also varied by province, with the highest proportion observed in Gilgit-Baltistan (37.1%) and the lowest in Sindh (17.5%). Household size appeared to play a role, as WRA from smaller families (<7 members) had a higher prevalence of B_12_ deficiencies (22.5%) compared to those from larger families (17.2%). Differences were also noted across various demographic and health-related factors. Women who were overweight or obese, had fewer number of parities, and were pregnant at the time of sample collection were more likely to be B_12_ deficient. However, all categories of age, education, food insecurity, wealth index, drinking water source, dietary diversity, sanitation facility, and anemia status showed no difference in the proportion of B_12_-deficient WRA.

Among all the samples analyzed for either folate or B_12_, there were 3986 samples that were tested for both micronutrients, thus permitting the assessment of concurrent deficiencies. In the total of those analyzed for both micronutrients, ~48% were found to have folate deficiency while ~21.5% were found to have B_12_ deficiency; therefore, they showed similar deficiencies corresponding to that of samples that were analyzed separately. In total, 10.3% of the WRA were found to have combined deficiencies in folate and B_12_, thereby indicating a significant prevalence of dual micronutrient inadequacy in this group.

Combined deficiencies were found in 11.8% of rural women, which is significantly higher than in urban women, who had a prevalence of 8.1%. A significant provincial variation was noted here as well, and the highest prevalence was in Baluchistan (26.5%), followed by the Islamabad Capital Territory (17.6%) and KP-NMD (18.0%). Other regions reported lower rates: Punjab (11.6%), Sindh (8.8%), KP (9.5%), AJK (12.8%), and Gilgit-Baltistan (12.0%).

Household size showed differences in proportions; a greater proportion of women from smaller families (<7 members) had combined deficiencies (11.7%) than those from larger families (≥7 members) (8.4%). No significant differences were found in the categories of wealth index, water source, sanitation, and food insecurity. Age, education, occupation, marital status, parity, minimum dietary diversity, and hemoglobin levels likewise did not indicate significant differences in the proportion of dual deficiencies.

Among physiological and health-related characteristics, BMI showed a difference in proportions, with combined deficiencies increasing from 7.1% in women classified as underweight, based on BMI (<18.5 kg/m^2^), to 13.7% in the case of women categorized as being obese (≥30 kg/m^2^), according to the same measure. The status of being pregnant was another important factor that appeared with different proportions of deficiencies, the prevalence of which was significantly higher among pregnant women (19.2%) than among non-pregnant women (9.7%).

### 3.3. Key Factors Associated with Folate and B_12_ Deficiency in Women of Reproductive Age

Table 3 presents the adjusted and unadjusted associations of covariates with folate and B_12_ deficiencies among women of reproductive age. At the univariate level, while analyzing for the association of individual factors, the odds of being folate deficient among WRA varied significantly across the categories of province, drinking water source, sanitation facility, food insecurity status, and age, while showing weak associations with wealth index and education levels. After adjusting for covariates in the model, the odds of being folate-deficient among WRA were not significantly associated with residence, food insecurity status, and wealth index. Significant association was observed for provincial distribution, that is, the odds of being folate deficient among WRA in Sindh, Baluchistan and Islamabad were observed to be 1.140 (95% CI 1.018, 1.285), 1.237 (95% CI 1.052, 1.453) and 1.524 (95% CI 1.109, 2.092) times compared to in Punjab, while Azad Jammu Kashmir (AJK) showed a moderate association with lower odds of being folate deficient, which was 0.834 (95% CI 0.682, 1.021) times, suggesting a 16.6% lower likelihood compared to Punjab.

The univariate analysis of B_12_ deficiency among WRA was observed to vary across the categories of residence, province, household size, wealth index, food insecurity status, age, education, BMI, parity, and pregnancy status. After adjusting for these covariates during multivariable analysis, the wealth index showed an insignificant association but was kept in the model, due to its theoretical and contextual relevance. Among the WRA in Pakistan, the odds of B_12_ deficiency were associated with 1.407 (95% CI 1.125, 1.760) times higher in rural areas compared to urban areas. Higher odds of being B_12_ deficient were associated with those living in Islamabad, Gilgit Baltistan, and newly merged districts of KPK, i.e., 1.673 (95% CI 1.122, 2.497), 2.472 (95% CI 1.197, 5.106), and 1.584 (95% CI 0.977, 2.570), respectively, while Sindh showed a moderate association with lower odds of being B_12_ deficient, which was 0.814 (95% CI 0.654, 1.013) times, suggesting an 18.6% lower likelihood compared to Punjab. The B_12_ deficiency was prevalent in households with <7 members (OR 1.385, 95% CI 1.152, 1.664), obese (OR 1.649, 95% CI 1.282, 2.122) and overweight (1.560, 95% CI 1.262, 1.928) WRA, those having less than three parities (OR 1.247, 95% CI 1.042, 1.492), and among pregnant women (OR 1.903, 95% CI 1.374, 2.635). Being B_12_-deficient was observed to be associated with 20.0%, 32.4%, and 20.5% lower odds in WRAs with mild, moderate, or severe food insecurity compared to those with no food insecurity. Surprisingly, the odds of being B_12_ deficient were 31.6% and 38.7% lesser in WRA with lower education levels (primary and secondary) compared to those with higher education. To further assess multicollinearity, variance inflation factors (VIF) were calculated for all independent variables. The mean VIF was 1.40 and 1.54, suggesting low multicollinearity and confirming the adequacy of the folate and B_12_ deficiency models.

### 3.4. Sensitivity Analysis for Food Insecurity and Vitamin B12 Deficiency

Table 4 illustrates the outcome of the stratified analysis by food security for evaluating factors related to B_12_ deficiency among Pakistani WRA. The analysis confirmed the initial regression results, as seen in Table 3, showing associations with the key predictor variables. Living in rural areas was strongly associated with both food-secure (aOR: 1.47; 95% CI: 1.07–2.02; *p* = 0.019) and food-insecure women (aOR: 1.41; 95% CI: 1.09–1.83; *p* = 0.009). Conversely, being a woman from a smaller family (<7 members) was still more likely to be associated with B_12_ deficiency (food-secure aOR: 1.64; 95% CI: 1.25–2.16; *p* < 0.001; food-insecure aOR: 1.58; 95% CI: 1.24–2.00; *p* < 0.001). Being overweight or obese, in addition to being pregnant, was persistent and strongly associated with higher odds of deficiency throughout both strata, like the previously observed patterns. There were noticeable differences between the provinces, but the associations among food-insecure women got weaker after controlling for other factors. Other variables like age, marital status, occupation status, dietary diversity, sanitation, and hemoglobin status were not found to have consistently significant associations in either group. The overall result of this sensitivity analysis showed that living in rural areas, having a smaller family size, having a higher BMI, and pregnancy are the factors associated with B_12_ deficiency in WRA, regardless of food security status.

## 4. Discussion

Folate and B_12_ analysis has been performed on a subset of blood samples collected from WRAs enrolled in the NNS Pakistan 2018. This analysis reflects nationally representative estimates of plasma B_12_ and folate deficiencies in Pakistan’s WRA population. The prevalence of folate deficiency (44.7%) was higher than that of B_12_ deficiency (20.2%), which was consistent with the pattern observed in NNS-2011. However, the prevalence of B_12_ deficiency was lower in NNS-2018 compared to NNS-2011 [19]. National-level nutrition intervention programs, like the Fortification Initiative Program (2016–2018), targeting wheat and edible oil fortification [34,35] may have contributed to the declining trend in B_12_ deficiency among WRA. However, these interventions included both folate and B_12_ fortification, but as studies suggest that folate [36] is less heat-stable than B_12_ [37] and can degrade during high-temperature cooking, this may likely contribute to disparity between trends of folate and B_12_ deficiency in the population, as in Pakistan, common wheat breads such as chapatis and naans are prepared on a hot skillet or oven and are often touched by open flames or walls of heated ovens (tandoors) for puffing, which potentially expose dough to thermal stress and could be one of the reasons leading to lower folate levels in the population [38]. There are also many other ongoing projects in Pakistan that are designed to tackle the issue of micronutrient deficiency, specifically among WRA, through different strategies like supplementation and nutrition-sensitive social protection programs. The evidence-based methods used to deal with the problem outlined in the Pakistan Maternal Nutrition Strategy (2022–2027) consist of the promotion of the intake of iron–folic acid and multiple micronutrient supplements (MMS) among pregnant women and breastfeeding mothers as part of the enlarged maternal nutrition services. Health sector collaboration is involved in the promotion of MMS, offering the essential minerals and vitamins such as iron and folic acid in one formulation, thus improving maternal nutrition beyond the regular iron–folic acid supplementation, to significantly raise the women’s coverage by 2027 [39]. The Benazir Nashonuma Programme (BNP), the flagship conditional cash transfer initiative of the Benazir Income Support Programme that officially launched in August 2020, complements these interventions specifically targeting nutrition by offering specialized nutritious supplementation, cash support, and behavior change assistance for pregnant and breastfeeding women to improve maternal and child nutrition outcomes, including decreases in stunting and health risks due to a lack of micronutrients in the target groups [40].

These estimates have been further evaluated for association with important geographical, demographic, socioeconomic, and health-related factors. Substantial variations in the prevalence of both micronutrients were observed at the provincial level. Yet, no disparity was evident between rural and urban populations, except for B_12_, which was significantly associated with higher odds of deficiency among rural populations. Research from geographically similar regions of South-East Asia suggests that folate deficiency continues to be a public health problem, affecting both rural and urban populations, with a slightly higher prevalence in rural populations, like studies conducted among adolescent girls and WRA in Iran, a developing country, and in Turkey, a relatively more developed Asian country, which report rural folate deficiency rates of 30.4% and 20.1%, respectively, compared to 32.7% and 14.7% in urban areas. While the pattern in Iran shows a marginally higher urban prevalence, the overall trend across countries leans toward a greater burden in rural populations [41,42]. Moreover, our findings showed no significant association of folate with food insecurity status, residence, and wealth index, but these were kept in the model due to their theoretical relevance [43,44,45]. These factors are established nutritional status indicators that may act as a confounder and distort the associations, thereby including them, resulting in more robust and conceptually sound models. The lack of observed associations in our study may be due to the residual confounding of unmeasured factors, including dietary patterns, supplementation practices, and cultural behaviors. Additionally, folate status may be more strongly influenced by individual-level factors, rather than broader socioeconomic indicators, in this setting, which could explain the absence of a detectable association.

The education level of WRA surprisingly had a positive association among the less educated, i.e., a lower risk of B_12_ deficiency compared with women with secondary and higher education. Our findings on socioeconomic factors are consistent with the model proposed by the HELENA study, a cross-sectional, multicenter study on nutrition and lifestyle among adolescents from 10 European cities in nine countries. The study focused on investigating socioeconomic determinants as predictors of folate and B_12_ intake and plasma levels, supporting the idea that SES, particularly maternal education and occupation, exerts an important influence on diet, affecting the blood’s vitamin and nutrient levels. However, genetic factors [46], physiological status, and their interactions with other nutrients can influence these levels as well [47]. Similar observations for the wealth index, a key factor of SES, have been observed to be positively associated with both micronutrients in Guatemala among WRA conducted in 2009–2010 (48). In contrast, our preliminary analysis showed an association with a higher likelihood of B_12_ deficiency in women of the richest households. To further explore this unexpected finding, a sensitivity analysis stratified by food security status was conducted. The results showed that women of both food-secure and food-insecure households from the richest wealth quintile had higher odds of B_12_ deficiency. This suggests that the observed association is unlikely to be explained solely by food security status. One possible explanation may be the increased consumption of processed or refined foods, displacing traditional diets that are rich in micronutrients among the richest households. Another potential factor could be the prevalence of overweight and obesity being around double in wealthier women in Pakistan [48], which can contribute to altered vitamin B_12_ metabolism and lower circulating levels, as is evident in some studies [49]. However, these interpretations should be considered cautiously, as detailed data related to dietary intake and lifestyle behaviors may also contribute to the observed association.

An analysis of findings from the Canadian Community Health Survey (CCHS 2.2) indicates a higher prevalence of nutrient inadequacy (including folate and B_12_) among adolescents and adults in food-insecure households [50]. However, in our analysis, the multivariable regression surprisingly showed an association of having lower odds of B_12_ deficiency among women facing mild, moderate, or even severe food insecurity compared to those with no food security, and less educated women (primary or secondary) seemed to be less prone to deficiency than highly educated women. The protective effect could be a result of residual confounding, measurement limitations, or reverse causality. The observed association of food insecurity with B_12_ deficiency could be due to unmeasured factors, including supplementation use, cultural dietary patterns, and access to fortified food programs. The combined effects of household-level food insecurity at a single time and a single serum B_12_ measurement can create challenges in estimating the individual long-term status. Some food-insecure households consume animal-source foods more than other foods, or they may have specific dietary patterns that result in a higher B_12_ intake, although this hypothesis remains unproven. Moreover, the food composition plays a more important role than the intake, as having food insecurity is not as associated with being micronutrient deficient, but may reflect variations in diet quality and food choices [51]. Finally, there could be a misclassification of food insecurity status and dietary intake, which could further bias the observed association.

To further assess whether the predictors of B_12_ deficiency differ between food-secure and food-insecure groups, and to clarify the effect of food insecurity on deficiency risk, these counterintuitive findings were analyzed by stratifying participants by their food security status. The sensitivity analysis generally shows that rural living, small family size, high BMI, and pregnancy are predictors that are positively associated with B_12_ deficiency among WRA, regardless of food security status, and elucidates the unexpected associations with food security that are observed in the initial regression (Table 3). These results reinforce the need for a cautious interpretation of the apparent protective association between food insecurity and B_12_ deficiency and suggest that other unmeasured contextual or behavioral factors may play a role. Similarly, a lower level of education was protective against B_12_ deficiency. This finding contrasts with much of the existing literature, which generally reports a higher prevalence of micronutrient deficiencies among individuals with lower levels of education. This can be due to factors such as the fact that in certain communities, women with less education live in rural areas, which enables them to consume more animal-sourced foods that local regions provide, and these foods serve as their main vitamin B_12_ sources. Second, education level may not fully capture nutritional knowledge or household food decision-making related to food choices. Therefore, these findings should be interpreted cautiously.

Among growth-related factors, we observed higher odds of B_12_ deficiency associated with overweight and obese WRA. These findings align with the existing literature, further supporting the association between excess body weight and an increased risk of B_12_ deficiency [52,53]. This association might be attributed to underlying physiological mechanisms, such as obesity predisposing them to alterations in their metabolic pathways, leading to malabsorption, increased catabolism, and sequestration in adipose tissue [54]. However, the basic cause of obesity is an inadequate or unbalanced diet, which tends to be associated with the intake of foods with high energy but lower diversity and the intake of B_12_-rich foods such as lean meats, fish, dairy products, and eggs [55]. In contrast, obese women are generally perceived as being healthier in settings with low awareness. A 2018 review article on the present and future attitudes towards obesity in Pakistan documented that nearly 52% of overweight and 73% of obese people do not perceive their condition as being abnormal [56], which may predispose them to not sense the requirement for micronutrient supplementation, resulting in a low B_12_ status. Moreover, in low-resource settings like Pakistan, where a lesser percentage of household spending is on milk, meat, and fruits [57], the risk of B_12_ deficiency in obese women can be even greater.

Age, an important demographic factor, was assessed qualitatively by categorizing the study population into specific age groups. Age was only found to be significantly associated with both folate and B_12_ deficiencies during the preliminary analysis. Younger groups of WRA, i.e., 15–19 years old, showed a higher association with folate deficiency, and those who were 20–34 years old showed a higher association with B_12_ deficiency compared to the older age group (35–49 years). Female adolescents and those in their twenties have higher metabolic activity and increased nutritional demands, due to ongoing physiological growth, menstruation, and possible pregnancies [58]. Thus, these increased requirements for essential micronutrients such as folate and B_12_ place younger WRA at a higher risk for deficiency. This was also evident in the last survey of Pakistan, in which younger WRA had 1.15 times the odds of being folate-deficient and 1.07 times the odds of being B_12_-deficient compared with older WRA; similarly, the association was insignificant after adjustment for other factors [19]. Similarly, no association was observed between folate or B_12_ deficiency and anemia, as measured by hemoglobin levels, regardless of household food security. This aligns with population-based surveys that suggest that in LMICs, anemia is a complex problem that is not solely a result of iron, folate, or B_12_ deficiencies. Moreover, hemoglobin is not a specific indicator and may not fall below normal, even in early or subclinical vitamin deficiency, as hematologic manifestations typically take a long time to develop. Potential contributors to anemia-like infections, inflammation, hemoglobin disorders, and an important biomarker, i.e., serum iron concentration, which are very common in these regions, may have independent effects on hemoglobin levels but were not measured primarily in the survey, which limited our capacity for the evaluation of anemia etiology in the population through a cross-sectional survey [59]. Therefore, anemia was not considered to be the primary outcome measure, since anemia is a multifactorial condition, and we primarily focused on assessing the association of B_12_ and folate deficiencies with a variety of sociodemographic factors and nutritional factors like anemia, because these micronutrient deficiencies have significant public health importance, as they can cause a variety of health problems, such as alterations in neurological functions, deoxyribonucleic acid (DNA) synthesis, and reproductive health [60]. The higher fertility rates associated with the younger WRA entail additional pregnancies and breastfeeding, which consequently drain maternal folate and B_12_ stores [61,62]. This may be one of the contributors to lower B_12_ levels in our study among pregnant women. These disparities may also be attributed to the increasing use of oral contraceptive pills (OCPs) in the population, which have been associated with lowering serum folate and B_12_ levels [63,64]. The increasing accessibility to affordable and diverse contraceptive options in the region is evident in the success rates of widespread free family planning campaigns [65,66,67]. All these factors may explain the higher likelihood of folate and B_12_ deficiencies among younger WRA compared to their older counterparts.

The two micronutrients serve as the vital components needed for red blood cell development, yet our study findings showed no link between anemia and micronutrient deficiencies in the adjusted model. These findings can be explained by multiple reasons; the most evident is that anemia in this population exists as a complex multifactorial condition, which can be influenced not only by folate and B_12_ levels, but can be due to iron deficiency, parasitic infections, chronic inflammation and genetic hemoglobin disorders and the current study lacks data on these other variables, limiting our understanding of the results presented. These findings are consistent with a study conducted by Bando et al. in 2023 [68], which discovered that approximately 10% of patients with normocytic anemia showed deficiencies in either folate or B_12_, despite lacking the usual macrocytic symptoms. The research demonstrated that vitamin replacement therapy resulted in hemoglobin improvement for a small number of cases, which indicates that normocytic anemia with these deficiencies depends on multiple factors that include preexisting medical conditions.

Additionally, the limitation of cross-sectional data prevents the researchers from establishing causal relationships, and single biochemical tests cannot show complete long-term micronutrient patterns that are relevant to hemoglobin synthesis. Moreover, factors like being pregnant, residing at a higher altitude, and lower hydration status can influence hemoglobin levels, which result in a less effective link between micronutrient deficiencies and anemia [69,70,71]. Despite the lack of a statistically significant association, it remains biologically plausible that folate and B_12_ deficiencies contribute to megaloblastic anemia, particularly in subgroups with coexisting iron deficiency.

To evaluate the prevalence of combined deficiencies of both vitamins and their distribution, we examined the occurrence of these micronutrients in the overlapping data from folate and B_12_ analyses. WRA having both folate and B_12_ deficiency was about 10.3%. This observed occurrence of combined deficiencies of folate and B_12_ underlines a major public health issue, mainly in the case of rural women, pregnant women, and women with higher BMI. The results indicate that remedies should not only target individual micronutrients but also consider dual supplementation to more effectively address overlapping deficiencies.

This research offers several significant strengths. First, it draws on data from the Pakistan National Nutrition Survey 2018, providing a large, nationally representative sample of WRA from various provinces and regions, which increases the generalizability of the results. Moreover, standardized biochemical measurements were used to assess the folate and B_12_ concentrations, ensuring that the indicators of the micronutrient status were reliable and objective. This study also included numerous sociodemographic and health-related factors, making it easier to understand the factors associated with deficiencies. Nevertheless, there are a few limitations to the study. The lack of definitive causality is one of the main limitations of this cross-sectional design, as this inherently limits the causal inference for the observed associations like those observed between drinking water source, BMI, or pregnancy status with the measured outcomes, and therefore, these cannot be interpreted as causal relationships. Moreover, the temporal sequence between exposure and outcome cannot be evaluated; consequently, residual confounding may influence the observed associations. The biochemical analysis was conducted only as a secondary analysis, limiting control over the design and sampling techniques. Moreover, due to the cross-sectional nature of the data and the relatively high prevalence of the outcome, the estimated odds ratios are considered measures of association, reflecting associations at a single point in time, rather than direct estimates of risk or incidence. However, logistic regression was used, due to its flexibility and widespread application. We acknowledge that prevalence ratios are more interpretable for common outcomes and, therefore, the findings should be interpreted with caution, particularly for outcomes showing a higher strength of association.

Additionally, the use of forward stepwise selection is also one of the limitations, as it may result in model instability and overfitting, and the findings should therefore be interpreted with caution. As the study was conducted in a single country, this limits the external validity of the findings when generalizing results to other settings. Another limitation of this study was the unequal sample sizes analyzed for folate and B_12_, along with disproportionate sample sizes from the provinces of Pakistan; for example, Gilgit-Baltistan and KP-NMD had a relatively small sample size. Therefore, the results for these provinces need to be viewed with caution, since the confidence intervals are very wide, suggesting that the results for these provinces are not precise or stable. The situation was caused by the analysis of archived biological samples, where the remaining amount of the sample limited the number of specimens, particularly for the analysis of B_12_, as the B_12_ assay requires a larger sample volume than the folate. As a result, more samples were taken for the folate measurements. For this analysis, we adopted a pragmatic, availability-based sampling approach. The folate and B_12_ analyses were conducted on all eligible archived plasma samples with sufficient residual volume. The study was conducted on a subset of participants without true random sampling from the original cohort, which could have introduced selection bias. However, testing all eligible samples minimizes the selection bias, and the large sample sizes support robust and reliable estimates. We acknowledge the limitation that this convenience-based subsample may not be fully representative of the overall study population. Other factors that have not been measured, for instance, the presence of genetic polymorphisms, cooking habits, and use of different micronutrient supplementations, might also affect folate and B_12_ levels. Moreover, one-time blood-level measurements do not accurately reflect the long-term nutrient status, and using 2018 data may limit the relevance of the findings in terms of timing, relative to the implementation of nutrition programs in the region.

The analysis process included various efforts aiming to decrease any potential bias sources that could affect the results. We used sampling weights to correct for the unequal participant distribution across different provinces, which enabled them to achieve better national-level estimation accuracy. Multivariable regression models enabled researchers to control the essential sociodemographic elements and health variables, which helped them to decrease the confounding variables. Biochemical analyses were conducted through standardized laboratory methods, which included quality control protocols to guarantee accurate measurement results for the folate and B_12_ levels. The researchers interpreted results from provinces that had smaller sample sizes with caution because the results produced wider confidence intervals, but still, the smaller analytical sample for B_12_ might have resulted in limited statistical power. The result of this study holds considerable implications for public health and policy. The study highlights the regions in Pakistan in which WRA suffers from folate and B_12_ deficiencies. Detailed raw data on food consumption may also help us to design targeted interventions, such as food fortification, micronutrient supplementation, and dietary counseling programs. Pointing out these disparities across regions and demographics helps policymakers to prioritize high-risk populations, optimize resource allocation, and consider region-specific targeted interventions. First, the existing wheat flour fortification programs need stronger monitoring to ensure the compliance of flour mills with the national fortification standards by flour mills. The next step should involve the establishment of better antenatal healthcare and reproductive health services, which should provide women with folic acid and multiple micronutrient supplements during their entire pregnancy, with the focus being on increasing and sustaining a high compliance rate. The third and most important step is to have community-based nutrition education programs on dietary counseling programs, which will teach people to eat locally available foods that contain folate and vitamin B_12_, such as leafy vegetables, dairy products, eggs, and animal-source foods.

In addition, the implementation of regular serological assessments of micronutrient levels within national nutrition monitoring systems is required to allow health authorities to observe folate and B_12_ status changes throughout different time periods. The assessments will also enable an evaluation of fortification and supplementation effectiveness, which will assist in advancing national nutrition strategies based on scientific evidence.

## 5. Conclusions

This study highlights that nearly half of Pakistani WRA had folate deficiency, while one in five had vitamin B_12_ deficiency. Regional, nutritional, and physiological factors, particularly obesity, rural residence, high fertility, and pregnancy, were key associated factors. Despite the national fortification programs, disparities in the population persist, which indicates gaps in policy implementation. Targeted interventions promoting dietary diversification, fortified foods, and age- and pregnancy-specific supplementation are essential. Other areas that remain salient for research include dietary patterns and the intake of bioavailable folate and B_12_, particularly in relation to food fortification and dietary diversification strategies.

## Figures and Tables

**Table 1:** Characteristics of the study population among women of reproductive age.

Characteristics	Folate (*n* = 12,662) Weighted % (95% CIs)	Vitamin B12 (*n* = 4442) Weighted % (95 CIs)
**Residence**		
Urban	40.0 (38.9–41.2)	40.0 (38.3–41.7)
Rural	60.0 (58.8–61.1)	60.0 (58.3–61.7)
**Province**		
Punjab	49.7 (48.6–50.8)	29.9 (28.3–31.6)
Sindh	32.8 (31.7–33.9)	60.4 (58.7–62.1)
KP	9.5 (9.0–10.0)	3.3 (2.8–3.9)
Baluchistan	4.1 (3.8–4.3)	1.8 (1.4–2.2)
ICT	1.2 (1.0–1.4)	2.5 (2.1–2.9)
KP-NMD	1.0 (0.9–1.1)	1.4 (1.1–1.7)
AJK	1.4 (1.3–1.5)	0.6 (0.5–0.8)
GB	0.4 (0.3–0.4)	0.1 (0.1–0.1)
**HH size**		
<7	56.9 (55.8–58.0)	57.3 (55.6–58.9)
7 or more members	43.1 (42.0–44.2)	42.7 (41.1–44.4)
**Wealth Index**		
Poorest	19.7 (18.9–20.5)	27.3 (25.8–28.8)
Second	19.1 (18.2–19.9)	18.9 (17.6–20.2)
Middle	20.3 (19.4–21.2)	18.8 (17.5–20.2)
Fourth	21.6 (20.7–22.6)	18.7 (17.4–20.1)
Richest	19.4 (18.5–20.3)	16.3 (15.0–17.6)
**Drinking water source**		
Improved sources	92.2 (91.5–92.9)	91.3 (90.2–92.2)
Unimproved sources	7.8 (7.1–8.5)	8.7 (7.8–9.8)
**Sanitation**		
Improved sanitation facility	83.5 (82.8–84.3)	76.7 (75.3–78.1)
Unimproved sanitation facility	16.5 (15.7–17.2)	23.3 (21.9–24.7)
**Food insecurity status**		
Food Secure	59.8 (58.7–60.9)	54.0 (52.4–55.7)
Mild food insecure	12.5 (11.7–13.2)	14.6 (13.5–15.9)
Moderate food insecure	8.4 (7.8–9.0)	10.0 (9.1–11.1)
Severe food insecure	19.4 (18.5–20.3)	21.3 (20.0–22.7)
**Age**		
15–19 years	3.4 (3.0–3.8)	3.4 (2.9–4.1)
20–34 years	63.2 (62.1–64.2)	62.9 (61.3–64.6)
35–49 years	33.5 (32.4–34.5)	33.7 (32.1–35.3)
**Education**		
None	55.7 (54.5–56.8)	63.2 (61.6–64.9)
Primary	11.5 (10.8–12.3)	10.4 (9.4–11.4)
Middle	9.0 (8.4–9.6)	6.9 (6.1–7.8)
Secondary	12.8 (12.1–13.7)	10.5 (9.4–11.6)
Higher	11.0 (10.3–11.7)	9.0 (8.1–10.0)
**Occupation**		
Employed	4.6 (4.2–5.1)	4.8 (4.2–5.6)
Unemployed	95.4 (94.9–95.8)	95.2 (94.4–95.8)
**Marital status**		
Currently married	93.3 (92.7–93.8)	93.9 (93.1–94.7)
Ever M=married	1.6 (1.4–2.0)	1.5 (1.1–1.9)
Unmarried	5.1 (4.6–5.6)	4.6 (4.0–5.4)
**BMI of women**		
Underweight (<18.5)	11.0 (10.4–11.7)	14.3 (13.2–15.6)
Normal (18.5–24.9)	45.9 (44.8–47.0)	46.1 (44.4–47.8)
Overweight (25.0–29.9)	26.9 (25.9–27.9)	24.8 (23.4–26.4)
Obese (≥30)	16.2 (15.4–17.0)	14.7 (13.5–15.9)
**Parity**		
<3	45.8 (44.7–46.9)	43.5 (41.8–45.2)
≥3	54.2 (53.1–55.3)	56.5 (54.8–58.2)
**Pregnancy status**		
Pregnant	6.3 (5.8–6.9)	6.3 (5.5–7.3)
Non-Pregnant	93.7 (93.1–94.2)	93.7 (92.7–94.5)
**Minimum dietary diversity**		
<5 food groups	74.7 (73.6–75.7)	80.2 (78.7–81.6)
≥5 food groups	25.3 (24.3–26.4)	19.8 (18.4–21.3)

BMI: body mass index; HH: household; KP: Khyber Pakhtunkhwa; ICT: Islamabad Capital Territory; KP-NMD: Khyber Pakhtunkhwa newly merged districts; AJK: Azad Jammu and Kashmir; and GB: Gilgit-Baltistan. *n* = number of samples, % = percentage, and CI = Confidence Interval.

**Table 2:** Prevalence of B_12_ and folate deficiency among women of reproductive age.

Characteristics	Folate (*n* = 12,662) Deficiency Weighted % (95% CIs)	*p*-Values	Vitamin B12 (*n* = 4442) Deficiency Weighted % (95% CIs)	*p*-Values	Folate and Vitamin B12 (*n* = 3986) Deficiency Weighted % (95% CIs)	*p*-Values
**Overall**	**44.8** **(43.7–45.9)**		**20.2 (18.9–21.6)**		**10.3 (9.2–11.5)**	
**Residence**						
Urban	45.3 (43.4–47.1)	0.485	17.8 (15.8–20.0)	0.005	8.1 (6.7–9.8)	0.001
Rural	44.5 (43.1–45.8)		21.8 (20.1–23.7)		11.8 (10.3–13.4)	
**Province**						
Punjab	43.4 (41.7–45.0)	<0.001	22.9 (20.2–25.8)	<0.001	11.6 (9.5–14.1)	<0.001
Sindh	46.9 (44.9–48.9)		17.5 (15.9–19.3)		8.8 (7.6–10.2)	
KP	43.5 (40.8–46.4)		22.4 (16.6–29.4)		9.5 (6.0–14.7)	
Baluchistan	48.7 (45.4–52.1)		31.8 (23.1–42.0)		26.5 (17.7–37.6)	
ICT	53.6 (45.9–61.1)		32.8 (25.8–40.6)		17.6 (12.0–25.0)	
KP-NMD	43.6 (36.3–51.2)		35.4 (26.4–45.6)		18.0 (11.0–28.0)	
AJK	39.1 (34.7–43.6)		26.5 (18.5–36.5)		12.8 (7.1–22.1)	
GB	40.7 (36.1–45.5)		37.1 (23.2–53.6)		12.0 (5.2–25.2)	
**HH size**						
<7	44.5 (43.0–46.0)	0.540	22.5 (20.7–24.4)	<0.001	11.7 (10.2–13.3)	0.004
7 or more members	45.2 (43.5–46.8)		17.2 (15.3–19.2)		8.4 (7.0–10.1)	
**Wealth Index**						
Poorest	45.5 (43.2–47.9)	0.652	18.3 (16.0–20.8)	0.088	10.3 (8.4–12.5)	0.693
Second	45.1 (42.8–47.5)		21.1 (18.2–24.4)		10.6 (8.3–13.4)	
Middle	44.9 (42.5–47.4)		22.7 (19.5–26.3)		11.5 (9.0–14.6)	
Fourth	45.4 (42.9–47.9)		17.9 (15.1–21.1)		8.8 (6.6–11.5)	
Richest	43.0 (40.3–45.6)		22.1 (18.8–25.7)		10.4 (8.1–13.3)	
**Drinking water source**						
Improved sources	44.5 (43.4–45.7)	0.189	20.3 (19.0–21.8)	0.553	10.2 (9.1–11.4)	0.547
Unimproved sources	47.7 (43.1–52.4)		18.7 (14.3–24.2)		11.5 (7.8–16.8)	
**Sanitation**						
Improved sanitation facility	44.4 (43.2–45.7)	0.132	20.4 (18.9–22.0)	0.534	10.1 (8.9–11.4)	0.432
Unimproved sanitation facility	46.6 (44.1–49.2)		19.4 (16.9–22.3)		11.1 (9.0–13.6)	
**Food insecurity status**						
Food secure	44.1 (42.7–45.5)	0.388	22.3 (20.4–24.2)	0.009	11.0 (9.5–12.6)	0.294
Mild food insecure	46.2 (43.1–49.4)		18.8 (15.6–22.5)		8.5 (6.2–11.6)	
Moderate food insecure	47.1 (43.0–51.2)		16.0 (12.5–20.3)		8.3 (5.7–11.8)	
Severe food insecure	45.1 (42.5–47.6)		17.8 (15.2–20.8)		10.6 (8.4–13.4)	
**Age**						
15–19 years	51.3 (45.3–57.3)	0.058	16.1 (10.2–24.5)	0.205	8.6 (3.9–17.7)	0.094
20–34 years	44.1 (42.7–45.5)		21.1 (19.4–22.9)		11.2 (9.9–12.8)	
35–49 years	45.4 (43.5–47.3)		19.0 (16.7–21.4)		8.7 (7.1–10.6)	
**Education**						
None	45.7 (44.2–47.1)	0.379	20.5 (18.8–22.3)	0.064	10.8 (9.5–12.3)	0.673
Primary	42.8 (39.5–46.2)		18.0 (14.5–22.1)		9.5 (6.9–13.1)	
Middle	44.9 (41.2–48.6)		18.4 (14.2–23.7)		8.5 (5.6–12.6)	
Secondary	44.3 (41.0–47.7)		17.3 (13.7–21.7)		8.9 (6.1–12.8)	
Higher	42.8 (39.5–46.2)		25.4 (20.9–30.4)		10.6 (7.4–14.9)	
**Occupation**						
Employed	44.0 (38.9–49.2)	0.757	20.0 (14.8–26.4)	0.936	8.6 (5.3–13.5)	0.435
Unemployed	44.8 (43.7–46.0)		20.2 (18.9–21.6)		10.4 (9.3–11.6)	
**Marital status**						
Currently married	44.8 (43.7–46.0)	0.979	20.2 (18.9–21.7)	0.723	10.4 (9.3–11.6)	0.482
Ever married	44.8 (36.3–53.6)		15.9 (8.2–28.5)		4.5 (1.1–16.9)	
Unmarried	44.3 (39.5–49.2)		21.0 (15.2–28.1)		10.8 (6.3–17.9)	
**BMI of women**						
Underweight (<18.5)	44.6 (41.4–47.8)	0.995	13.3 (10.7–16.5)	<0.001	7.1 (5.1–9.8)	0.009
Normal (18.5–24.9)	44.9 (43.2–46.5)		18.3 (16.5–20.3)		9.7 (8.2–11.4)	
Overweight (25.0–29.9)	44.9 (42.7–47.1)		24.6 (21.7–27.7)		11.3 (9.2–13.8)	
Obese (≥30)	44.5 (41.8–47.3)		25.5 (21.9–29.5)		13.7 (10.7–17.2)	
**Parity**						
<3	44.7 (43.1–46.4)	0.946	22.3 (20.3–24.5)	0.007	11.0 (9.3–12.8)	0.286
≥3	44.8 (43.3–46.3)		18.5 (16.9–20.4)		9.8 (8.4–11.3)	
**Pregnancy status**						
Pregnant	44.0 (39.5–48.6)	0.732	32.2 (25.9–39.3)	<0.001	19.2 (13.8–26.0)	<0.001
Non-Pregnant	44.8 (43.7–46.0)		19.4 (18.1–20.8)		9.7 (8.6–10.8)	
**Minimum dietary diversity**						
<5 food groups	45.3 (43.9–46.7)	0.320	19.4 (18.1–20.8)	0.811	11.0 (9.7–12.5)	0.298
≥5 food groups	43.9 (41.5–46.3)		20.6 (17.6–24.0)		9.4 (7.2–12.3)	
**Hb level**						
Anemic	45.2 (43.5–46.9)	0.594	20.6 (18.6–22.8)	0.646	10.6 (9.0–12.5)	0.609
Non-anemic	44.6 (43.1–46.0)		19.9 (18.2–21.8)		10.0 (8.7–11.5)	

Hb: hemoglobin; HH: household; BMI: body mass index; KP: Khyber Pakhtunkhwa; ICT: Islamabad Capital Territory; KP-NMD: Khyber Pakhtunkhwa Newly Merged Districts; AJK: Azad Jammu and Kashmir; and GB: Gilgit-Baltistan. Chi-Squared test was used to determine the statistical significance between folate and B_12_ deficiency between each category; *p* < 0.05 was considered significant.

**Table 3:** Multivariable ORs and 95% CIs of folate and B_12_ deficiency among women of reproductive age.

	Folate Deficiency				B_12_ Deficiency			
Characteristics	OR (95% CIs)	*p*-Values	aOR * (95% CIs)	*p*-Values	OR (95% CIs)	*p*-Values	aOR * (95% CIs)	*p*-Values
**Residence**								
Urban	Ref.		Ref.		Ref.		Ref.	
Rural	0.967 (0.881, 1.062)	0.485	0.967 (0.862, 1.085)	0.569	1.293 (1.083, 1.544)	0.005	1.407 (1.125, 1.760)	0.003
**Province**								
Punjab	Ref.		Ref.		Ref.		Ref.	
Sindh	1.152 (1.037, 1.280)	0.009	1.144 (1.018, 1.285)	0.024	0.717 (0.590, 0.872)	0.001	0.814 (0.654, 1.013)	0.065
KP	1.007 (0.881, 1.150)	0.921	1.003 (0.875, 1.149)	0.970	0.971 (0.651, 1.449)	0.887	0.953 (0.632, 1.437)	0.818
Baluchistan	1.241 (1.069, 1.440)	0.005	1.237 (1.052, 1.453)	0.010	1.575 (0.988, 2.510)	0.056	1.380 (0.857, 2.222)	0.184
ICT	1.505 (1.099, 2.062)	0.011	1.524 (1.109, 2.092)	0.009	1.643 (1.130, 2.389)	0.009	1.673 (1.122, 2.497)	0.012
KP-NMD	1.009 (0.738, 1.378)	0.957	1.005 (0.732, 1.379)	0.975	1.849 (1.176, 2.907)	0.008	1.584 (0.977, 2.570)	0.062
AJK	0.837 (0.686, 1.022)	0.081	0.834 (0.682, 1.021)	0.079	1.216 (0.743, 1.989)	0.436	1.296 (0.770, 2.182)	0.329
GB	0.897 (0.730, 1.103)	0.304	0.885 (0.715, 1.095)	0.261	1.990 (0.998, 3.966)	0.051	2.472 (1.197, 5.106)	0.015
**HH size**								
<7	0.972 (0.889, 1.064)	0.540			1.399 (1.175, 1.665)	<0.001	1.385 (1.152, 1.664)	0.001
7 or more members	Ref.				Ref.		Ref.	
**Wealth index**								
Poorest	Ref.		Ref.		Ref.		Ref.	
Second	0.984 (0.861, 1.125)	0.813	1.027 (0.892, 1.183)	0.708	1.200 (0.935, 1.539)	0.152	1.064 (0.817, 1.386)	0.646
Middle	0.977 (0.852, 1.120)	0.739	1.022 (0.875, 1.195)	0.781	1.317 (1.023, 1.695)	0.032	1.189 (0.894, 1.580)	0.234
Fourth	0.994 (0.866, 1.141)	0.929	1.031 (0.871, 1.221)	0.719	0.978 (0.754, 1.270)	0.870	0.901 (0.650, 1.248)	0.530
Richest	0.902 (0.782, 1.040)	0.156	0.924 (0.770, 1.110)	0.399	1.266 (0.976, 1.641)	0.075	1.155 (0.794, 1.680)	0.451
**Drinking water source**								
Improved sources	0.879 (0.726, 1.065)	0.189			1.107 (0.792, 1.546)	0.553		
Unimproved sources	Ref.				Ref.			
**Sanitation**								
Improved sanitation facility	0.916 (0.816, 1.027)	0.132			1.065 (0.874, 1.298)	0.534		
Unimproved sanitation facility	Ref.				Ref.			
**Food insecurity status**								
Food secure	Ref.		Ref.		Ref.		Ref.	
Mild food insecure	1.091 (0.949, 1.254)	0.223	1.072 (0.931, 1.234)	0.333	0.809 (0.630, 1.040)	0.098	0.760 (0.584, 0.991)	0.043
Moderate food insecure	1.128 (0.949, 1.342)	0.172	1.104 (0.924, 1.318)	0.276	0.667 (0.489, 0.909)	0.010	0.676 (0.492, 0.930)	0.016
Severe food insecure	1.040 (0.924, 1.171)	0.512	1.007 (0.885, 1.146)	0.913	0.758 (0.608, 0.943)	0.013	0.795 (0.627, 1.007)	0.057
**Age**								
15–19 years	1.267 (0.984, 1.632)	0.066			0.818 (0.473, 1.414)	0.471		
20–34 years	0.951 (0.865, 1.046)	0.300			1.142 (0.951, 1.372)	0.156		
35–49 years	Ref.				Ref.			
**Education**								
None	1.123 (0.967, 1.304)	0.129			0.757 (0.576, 0.995)	0.046	0.919 (0.662, 1.275)	0.613
Primary	0.998 (0.822, 1.212)	0.983			0.645 (0.449, 0.925)	0.017	0.684 (0.461, 1.014)	0.058
Middle	1.086 (0.888, 1.330)	0.421			0.664 (0.444, 0.994)	0.047	0.677 (0.444, 1.031)	0.069
Secondary	1.062 (0.876, 1.287)	0.541			0.616 (0.424, 0.895)	0.011	0.613 (0.421, 0.892)	0.011
Higher	Ref.				Ref.		Ref.	
**Occupation**								
Employed	0.966 (0.779, 1.199)	0.757			0.985 (0.678, 1.430)	0.936		
Unemployed	Ref.				Ref.			
**Marital status**								
Currently married	1.021 (0.835, 1.250)	0.836			0.956 (0.642, 1.425)	0.826		
Ever married	1.019 (0.681, 1.527)	0.926			0.712 (0.307, 1.652)	0.429		
Unmarried	Ref.				Ref.			
**BMI of women**								
Underweight (<18.5)	0.988 (0.854, 1.142)	0.868			0.688 (0.521, 0.909)	0.008	0.711 (0.534, 0.946)	0.019
Normal (18.5–24.9)	Ref.				Ref.		Ref.	
Overweight (25.0–29.9)	1.001 (0.897, 1.117)	0.986			1.456 (1.184, 1.790)	<0.001	1.560 (1.262, 1.928)	<0.001
Obese (≥30)	0.986 (0.866, 1.124)	0.837			1.530 (1.207, 1.940)	<0.001	1.649 (1.282, 2.122)	<0.001
**Parity**								
<3	0.997 (0.911, 1.091)	0.946			1.263 (1.067, 1.496)	0.007	1.247 (1.042, 1.492)	0.016
≥3	Ref.				Ref.		Ref.	
**Pregnancy status**								
Pregnant	0.967 (0.799, 1.171)	0.732			1.976 (1.436, 2.720)	<0.001	1.903 (1.374, 2.635)	<0.001
Non-Pregnant	Ref.				Ref.			
**Minimum dietary diversity**								
<5 food groups	Ref.				Ref.			
≥5 food groups	0.944 (0.844, 1.057)	0.320			1.027 (0.823, 1.283)	0.811		

aOR *: adjusted odds ratio; the adjusted odds ratios were derived from a mixed-effect logistic regression model and were adjusted to account for all the variables listed in the table that had a *p*-value less than 0.25 in the univariate analysis. A *p*-value < 0.05 was considered significant for multivariate analysis. HH: household; BMI: body mass index; KP: Khyber Pakhtunkhwa; ICT: Islamabad Capital Territory; KP-NMD: Khyber Pakhtunkhwa Newly Merged Districts; AJK: Azad Jammu and Kashmir; and GB: Gilgit-Baltistan.

**Table 4:** Sensitivity analysis for food insecurity and vitamin B12 deficiency.

	Food Secure				Food Insecure			
Characteristics	OR (95% CIs)	*p*-Values	aOR * (95% CIs)	*p*-Values	OR (95% CIs)	*p*-Values	aOR * (95% CIs)	*p*-Values
**Residence**								
Urban	Ref.		Ref.		Ref.		Ref.	
Rural	1.368 (1.091, 1.715)	0.007	1.467 (1.066, 2.018)	0.019	1.355 (1.005, 1.827)	0.046	1.411 (1.091, 1.826)	0.009
**Province**								
Punjab	Ref.		Ref.		Ref.		Ref.	
Sindh	0.669 (0.517, 0.866)	0.002	0.678 (0.493, 0.932)	0.017	0.801 (0.592, 1.083)	0.150	0.766 (0.577, 1.016)	0.065
KP	1.045 (0.640, 1.708)	0.859	1.053 (0.610, 1.818)	0.852	0.814 (0.402, 1.646)	0.566	1.134 (0.685, 1.878)	0.625
Baluchistan	1.588 (0.849, 2.970)	0.148	1.468 (0.716, 3.011)	0.294	1.574 (0.778, 3.187)	0.207	1.703 (0.880, 3.296)	0.114
ICT	1.225 (0.740, 2.026)	0.430	1.256 (0.685, 2.303)	0.462	2.449 (1.396, 4.299)	0.002	1.217 (0.718, 2.064)	0.465
KP-NMD	1.329 (0.749, 2.360)	0.331	1.402 (0.764, 2.574)	0.276	3.173 (1.506, 6.688)	0.002	1.356 (0.760, 2.420)	0.303
AJK	0.806 (0.426, 1.526)	0.507	1.049 (0.538, 2.044)	0.888	2.555 (1.168, 5.589)	0.019	0.896 (0.468, 1.714)	0.740
GB	1.888 (0.647, 5.508)	0.245	1.147 (0.306, 4.303)	0.839	2.315 (0.936, 5.728)	0.069	2.053 (0.670, 6.292)	0.208
**HH size**								
<7	1.439 (1.142, 1.814)	0.002	1.640 (1.247, 2.155)	<0.001	1.310 (1.005, 1.708)	0.046	1.575 (1.239, 2.001)	<0.001
7 or more members	Ref.		Ref.		Ref.		Ref.	
**Wealth index**								
Poorest	Ref.		Ref.		Ref.			
Second	1.315 (0.905, 1.909)	0.151	1.205 (0.782, 1.857)	0.397	1.038 (0.730, 1.475)	0.835		
Middle	1.577 (1.088, 2.286)	0.016	1.634 (1.058, 2.524)	0.027	0.971 (0.672, 1.401)	0.874		
Fourth	0.985 (0.681, 1.424)	0.934	1.058 (0.654, 1.712)	0.818	0.861 (0.567, 1.308)	0.483		
Richest	1.193 (0.835, 1.706)	0.332	1.216 (0.725, 2.039)	0.459	1.260 (0.773, 2.055)	0.354		
**Drinking water source**								
Improved sources	2.041 (1.107, 3.764)	0.022	2.205 (1.116, 4.356)	0.023	0.683 (0.453, 1.029)	0.068	2.384 (1.261, 4.509)	0.008
Unimproved sources	Ref.		Ref.		Ref.		Ref.	
**Sanitation**								
Improved sanitation facility	1.111 (0.814, 1.515)	0.507			0.907 (0.692, 1.188)	0.477		
Unimproved sanitation facility	Ref.				Ref.			
**Age**								
15–19 years	0.870 (0.445, 1.701)	0.684			0.755 (0.295, 1.933)	0.558		
20–34 years	1.174 (0.918, 1.500)	0.201			1.089 (0.826, 1.436)	0.547		
35–49 years	Ref.				Ref.			
**Education**								
None	0.791 (0.581, 1.077)	0.137	0.738 (0.492, 1.107)	0.142	1.433 (0.695, 2.954)	0.330		
Primary	0.609 (0.397, 0.935)	0.023	0.461 (0.269, 0.791)	0.005	1.318 (0.582, 2.988)	0.508		
Middle	0.566 (0.348, 0.920)	0.022	0.383 (0.212, 0.694)	0.002	1.567 (0.658, 3.734)	0.310		
Secondary	0.517 (0.334, 0.799)	0.003	0.455 (0.279, 0.743)	0.002	1.536 (0.657, 3.590)	0.322		
Higher	Ref.		Ref.		Ref.			
**Occupation**								
Employed	1.272 (0.775, 2.089)	0.341			0.838 (0.472, 1.490)	0.548		
Unemployed	Ref.				Ref.			
**Marital Status**								
Currently married	0.928 (0.564, 1.525)	0.767			1.056 (0.537, 2.074)	0.875		
Ever married	0.729 (0.215, 2.473)	0.612			0.854 (0.266, 2.744)	0.791		
Unmarried	Ref.				Ref.			
**BMI of women**								
Underweight (<18.5)	0.728 (0.496, 1.069)	0.105	0.766 (0.497, 1.179)	0.226	0.665 (0.443, 0.999)	0.049	0.773 (0.523, 1.142)	0.196
Normal (18.5–24.9)	Ref.		Ref.		Ref.		Ref.	
Overweight (25.0–29.9)	1.440 (1.097, 1.890)	0.009	1.624 (1.185, 2.224)	0.003	1.425 (1.035, 1.963)	0.030	1.602 (1.211, 2.118)	0.001
Obese (≥30)	1.512 (1.116, 2.048)	0.008	1.671 (1.173, 2.381)	0.005	1.485 (1.009, 2.186)	0.045	1.614 (1.182, 2.204)	0.003
**Parity**								
<3	1.103 (0.884, 1.378)	0.386			1.454 (1.120, 1.888)	0.005		
≥3	Ref.				Ref.			
**Pregnancy status**								
Pregnant	1.988 (1.258, 3.141)	0.003	2.055 (1.241, 3.403)	0.005	2.121 (1.358, 3.313)	0.001	1.953 (1.213, 3.145)	0.006
Non-pregnant	Ref.		Ref.		Ref.		Ref.	
**Minimum dietary diversity**								
<5 food groups	Ref.		Ref.		Ref.			
≥5 food groups	0.924 (0.705, 1.211)	0.565	0.770 (0.579, 1.026)	0.074	1.074 (0.714, 1.617)	0.731		
**Hb level**								
Anemic	0.965 (0.768, 1.212)	0.760			1.208 (0.931, 1.567)	0.154		
Non-anemic	Ref.				Ref.			

aOR *: adjusted odds ratio; the adjusted odds ratios were derived from a logistic regression model and were adjusted to account for all the variables listed in the table that had a *p*-value less than 0.25 in the univariate analysis. A *p*-value ≤ 0.10 was considered significant for multivariate analysis. Hb: hemoglobin; HH: household; BMI, body mass index; KP: Khyber Pakhtunkhwa; ICT: Islamabad Capital Territory; KP-NMD: Khyber Pakhtunkhwa Newly Merged Districts; AJK: Azad Jammu and Kashmir; and GB: Gilgit-Baltistan.

## Data Availability

The raw data supporting the conclusions of this article will be made available by the corresponding author upon request.
